# Supplementary material for: TLE3 loss confers AR inhibitor resistance by facilitating GR-mediated human prostate cancer cell growth
Source: eLife. 2019 Dec 19;8:e47430. doi: 10.7554/eLife.47430 (PMC6968917; doi:10.7554/eLife.47430)
Supplement: Supplementary file 1. — Motif enrichment analysis showing sequence motifs that are enriched at genes differentially expressed in enzalutamide-treated control cells compared to TLE3KO cells. [file elife-47430-supp1.pdf]

Homer Known Motif Enrichment Results (./motifDiscoveryAR/)

Homer *de novo* Motif Results
Gene Ontology Enrichment Results
Known Motif Enrichment Results (txt file)
Total Target Sequences = 1237, Total Background Sequences = 48542

Rank	Motif	Name	P-value	log P-pvalue	q-value (Benjamini)	# Target Sequences with Motif	% of Targets Sequences with Motif	# Background Sequences with Motif	% of Background Sequences with Motif	Motif File	SVG
1	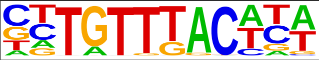	Foxa2(Forkhead)/Liver-Foxa2-ChIP-Seq(GSE25694)/Homer	1e-85	-1.962e+02	0.0000	660.0	53.35%	13057.7	26.90%	motif file (matrix)	svg
2	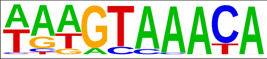	FOXA1(Forkhead)/LNCAP-FOXA1-ChIP-Seq(GSE27824)/Homer	1e-84	-1.946e+02	0.0000	887.0	71.71%	21464.1	44.22%	motif file (matrix)	svg
3	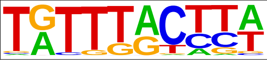	FOXM1(Forkhead)/MCF7-FOXM1-ChIP-Seq(GSE72977)/Homer	1e-80	-1.851e+02	0.0000	804.0	65.00%	18525.1	38.16%	motif file (matrix)	svg
4	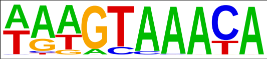	FOXA1(Forkhead)/MCF7-FOXA1-ChIP-Seq(GSE26831)/Homer	1e-80	-1.845e+02	0.0000	812.0	65.64%	18849.5	38.83%	motif file (matrix)	svg
5	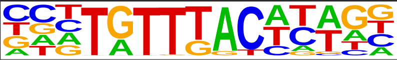	Foxa3(Forkhead)/Liver-Foxa3-ChIP-Seq(GSE77670)/Homer	1e-77	-1.777e+02	0.0000	400.0	32.34%	5872.3	12.10%	motif file (matrix)	svg
6	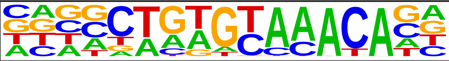	Fox:Ebox(Forkhead,bHLH)/Panc1-Foxa2-ChIP-Seq(GSE47459)/Homer	1e-76	-1.771e+02	0.0000	652.0	52.71%	13365.9	27.53%	motif file (matrix)	svg
7	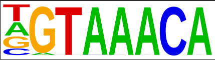	Foxo3(Forkhead)/U2OS-Foxo3-ChIP-Seq(E-MTAB-2701)/Homer	1e-52	-1.217e+02	0.0000	583.0	47.13%	12929.4	26.63%	motif file (matrix)	svg
8	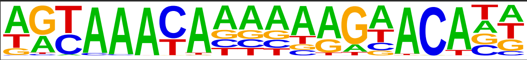	FOXA1:AR(Forkhead,NR)/LNCAP-AR-ChIP-Seq(GSE27824)/Homer	1e-52	-1.205e+02	0.0000	174.0	14.07%	1717.7	3.54%	motif file (matrix)	svg
9	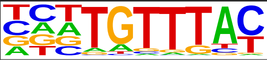	FOXK1(Forkhead)/HEK293-FOXK1-ChIP-Seq(GSE51673)/Homer	1e-51	-1.192e+02	0.0000	705.0	56.99%	17325.1	35.69%	motif file (matrix)	svg
10	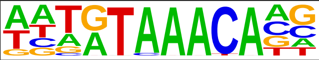	FoxL2(Forkhead)/Ovary-FoxL2-ChIP-Seq(GSE60858)/Homer	1e-45	-1.057e+02	0.0000	665.0	53.76%	16444.6	33.88%	motif file (matrix)	svg
11	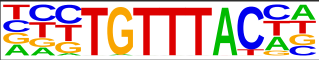	FOXP1(Forkhead)/H9-FOXP1-ChIP-Seq(GSE31006)/Homer	1e-45	-1.040e+02	0.0000	393.0	31.77%	7549.7	15.55%	motif file (matrix)	svg
12	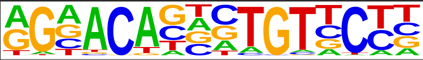	ARE(NR)/LNCAP-AR-ChIP-Seq(GSE27824)/Homer	1e-43	-1.004e+02	0.0000	190.0	15.36%	2341.7	4.82%	motif file (matrix)	svg
13	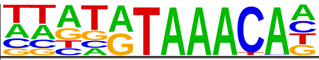	Foxf1(Forkhead)/Lung-Foxf1-ChIP-Seq(GSE77951)/Homer	1e-41	-9.550e+01	0.0000	696.0	56.27%	18054.7	37.19%	motif file (matrix)	svg
14	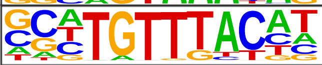	FOXK2(Forkhead)/U2OS-FOXK2-ChIP-Seq(E-MTAB-2204)/Homer	1e-40	-9.214e+01	0.0000	457.0	36.94%	9930.7	20.46%	motif file (matrix)	svg
15	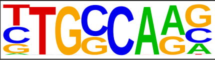	NF1-halfsite(CTF)/LNCaP-NF1-ChIP-Seq(Unpublished)/Homer	1e-38	-8.971e+01	0.0000	680.0	54.97%	17741.0	36.55%	motif file (matrix)	svg
16	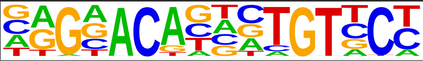	GRE(NR),IR3/A549-GR-ChIP-Seq(GSE32465)/Homer	1e-35	-8.280e+01	0.0000	121.0	9.78%	1196.9	2.47%	motif file (matrix)	svg
17	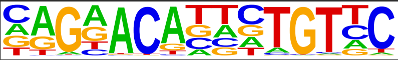	GRE(NR),IR3/RAW264.7-GRE-ChIP-Seq(Unpublished)/Homer	1e-35	-8.180e+01	0.0000	182.0	14.71%	2504.0	5.16%	motif file (matrix)	svg
18	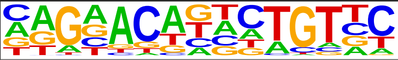	PR(NR)/T47D-PR-ChIP-Seq(GSE31130)/Homer	1e-32	-7.562e+01	0.0000	824.0	66.61%	24098.9	49.64%	motif file (matrix)	svg
19	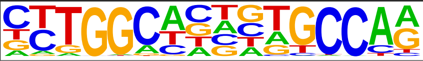	NF1(CTF)/LNCAP-NF1-ChIP-Seq(Unpublished)/Homer	1e-31	-7.322e+01	0.0000	224.0	18.11%	3743.3	7.71%	motif file (matrix)	svg
20	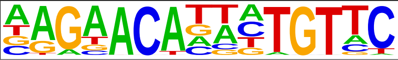	PGR(NR)/EndoStromal-PGR-ChIP-Seq(GSE69539)/Homer	1e-30	-7.062e+01	0.0000	200.0	16.17%	3192.7	6.58%	motif file (matrix)	svg
21	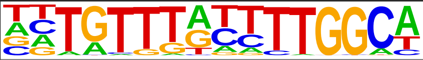	NF1:FOXA1(CTF,Forkhead)/LNCAP-FOXA1-ChIP-Seq(GSE27824)/Homer	1e-23	-5.448e+01	0.0000	82.0	6.63%	832.7	1.72%	motif file (matrix)	svg
22	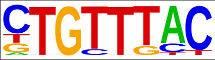	Foxo1(Forkhead)/RAW-Foxo1-ChIP-Seq(Fan_et_al.)/Homer	1e-23	-5.309e+01	0.0000	778.0	62.89%	23652.4	48.72%	motif file (matrix)	svg
23	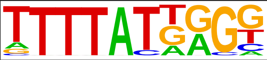	HOXB13(Homeobox)/ProstateTumor-HOXB13-ChIP-Seq(GSE56288)/Homer	1e-21	-4.953e+01	0.0000	640.0	51.74%	18540.3	38.19%	motif file (matrix)	svg
24	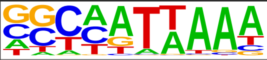	Unknown(Homeobox)/Limb-p300-ChIP-Seq/Homer	1e-15	-3.485e+01	0.0000	445.0	35.97%	12457.3	25.66%	motif file (matrix)	svg
25	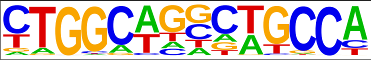	Tlx?(NR)/NPC-H3K4me1-ChIP-Seq(GSE16256)/Homer	1e-14	-3.431e+01	0.0000	182.0	14.71%	3858.9	7.95%	motif file (matrix)	svg
26	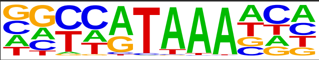	CDX4(Homeobox)/ZebrafishEmbryos-Cdx4.Myc-ChIP-Seq(GSE48254)/Homer	1e-14	-3.340e+01	0.0000	565.0	45.68%	16926.8	34.87%	motif file (matrix)	svg
27	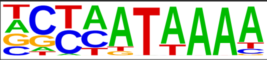	HOXD13(Homeobox)/Chicken-Hoxd13-ChIP-Seq(GSE38910)/Homer	1e-12	-2.902e+01	0.0000	675.0	54.57%	21495.4	44.28%	motif file (matrix)	svg
28	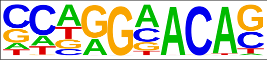	AR-halfsite(NR)/LNCaP-AR-ChIP-Seq(GSE27824)/Homer	1e-11	-2.725e+01	0.0000	959.0	77.53%	33273.8	68.54%	motif file (matrix)	svg
29	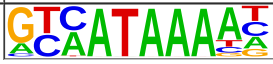	Cdx2(Homeobox)/mES-Cdx2-ChIP-Seq(GSE14586)/Homer	1e-9	-2.285e+01	0.0000	480.0	38.80%	14710.2	30.30%	motif file	svg

										(matrix)	
30	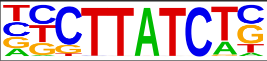	Gata2(Zf)/K562-GATA2-ChIP-Seq(GSE18829)/Homer	1e-8	-1.998e+01	0.0000	329.0	26.60%	9545.2	19.66%	motif file (matrix)	svg
31	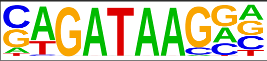	Gata1(Zf)/K562-GATA1-ChIP-Seq(GSE18829)/Homer	1e-8	-1.969e+01	0.0000	293.0	23.69%	8316.7	17.13%	motif file (matrix)	svg
32	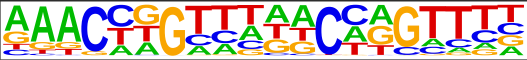	GRHL2(CP2)/HBE-GRHL2-ChIP-Seq(GSE46194)/Homer	1e-6	-1.574e+01	0.0000	182.0	14.71%	4871.8	10.04%	motif file (matrix)	svg
33	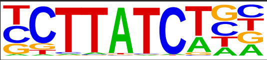	Gata6(Zf)/HUG1N-GATA6-ChIP-Seq(GSE51936)/Homer	1e-6	-1.568e+01	0.0000	441.0	35.65%	14022.3	28.89%	motif file (matrix)	svg
34	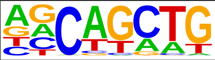	SCL(bHLH)/HPC7-Scl-ChIP-Seq(GSE13511)/Homer	1e-5	-1.378e+01	0.0000	972.0	78.58%	35274.9	72.67%	motif file (matrix)	svg
35	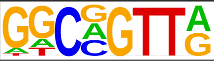	MYB(HTH)/ERMYB-Myb-ChIPSeq(GSE22095)/Homer	1e-5	-1.366e+01	0.0000	580.0	46.89%	19523.0	40.22%	motif file (matrix)	svg
36	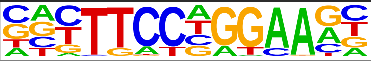	Stat3+il21(Stat)/CD4-Stat3-ChIP-Seq(GSE19198)/Homer	1e-5	-1.338e+01	0.0000	263.0	21.26%	7845.4	16.16%	motif file (matrix)	svg
37	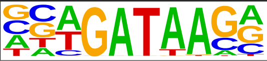	Gata4(Zf)/Heart-Gata4-ChIP-Seq(GSE35151)/Homer	1e-5	-1.331e+01	0.0000	459.0	37.11%	14981.8	30.86%	motif file (matrix)	svg
38	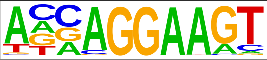	ELF3(ETS)/PDAC-ELF3-ChIP-Seq(GSE64557)/Homer	1e-5	-1.258e+01	0.0000	281.0	22.72%	8566.0	17.65%	motif file (matrix)	svg
39	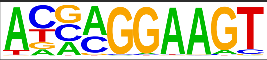	ELF5(ETS)/T47D-ELF5-ChIP-Seq(GSE30407)/Homer	1e-5	-1.252e+01	0.0000	276.0	22.31%	8394.1	17.29%	motif file (matrix)	svg
40	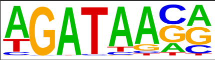	GATA3(Zf)/iTreg-Gata3-ChIP-Seq(GSE20898)/Homer	1e-4	-1.087e+01	0.0002	649.0	52.47%	22609.6	46.58%	motif file (matrix)	svg
41	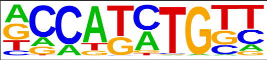	Olig2(bHLH)/Neuron-Olig2-ChIP-Seq(GSE30882)/Homer	1e-4	-1.077e+01	0.0002	623.0	50.36%	21611.6	44.52%	motif file (matrix)	svg
42	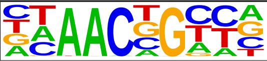	BMYB(HTH)/Hela-BMYB-ChIP-Seq(GSE27030)/Homer	1e-4	-1.053e+01	0.0002	527.0	42.60%	17948.0	36.97%	motif file (matrix)	svg
43	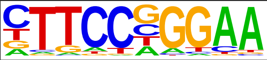	Stat3(Stat)/mES-Stat3-ChIP-Seq(GSE11431)/Homer	1e-4	-1.004e+01	0.0004	176.0	14.23%	5149.8	10.61%	motif file (matrix)	svg
44	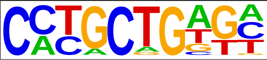	Zic(Zf)/Cerebellum-ZIC1.2-ChIP-Seq(GSE60731)/Homer	1e-4	-9.410e+00	0.0007	244.0	19.73%	7608.2	15.67%	motif file (matrix)	svg
45	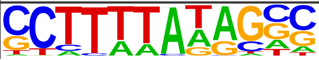	TATA-Box(TBP)/Promoter/Homer	1e-4	-9.374e+00	0.0007	555.0	44.87%	19207.3	39.57%	motif file	svg

										(matrix)	
46	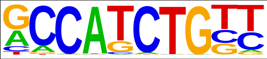	NeuroD1(bHLH)/Islet-NeuroD1-ChIP-Seq(GSE30298)/Homer	1e-3	-8.889e+00	0.0011	259.0	20.94%	8212.3	16.92%	motif file (matrix)	svg
47	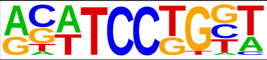	SPDEF(ETS)/VCaP-SPDEF-ChIP-Seq(SRA014231)/Homer	1e-3	-8.551e+00	0.0015	324.0	26.19%	10629.2	21.90%	motif file (matrix)	svg
48	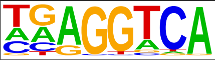	THRb(NR)/Liver-NR1A2-ChIP-Seq(GSE52613)/Homer	1e-3	-8.528e+00	0.0015	977.0	78.98%	36233.1	74.64%	motif file (matrix)	svg
49	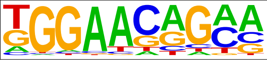	ZNF189(Zf)/HEK293-ZNF189.GFP-ChIP-Seq(GSE58341)/Homer	1e-3	-7.869e+00	0.0028	269.0	21.75%	8713.8	17.95%	motif file (matrix)	svg
50	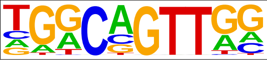	AMYB(HTH)/Testes-AMYB-ChIP-Seq(GSE44588)/Homer	1e-3	-7.785e+00	0.0030	498.0	40.26%	17297.1	35.63%	motif file (matrix)	svg
51	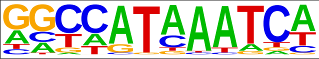	HOXA9(Homeobox)/HSC-Hoxa9-ChIP-Seq(GSE33509)/Homer	1e-3	-7.735e+00	0.0031	342.0	27.65%	11420.3	23.53%	motif file (matrix)	svg
52	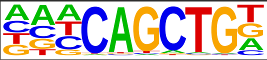	Ap4(bHLH)/AML-Tfap4-ChIP-Seq(GSE45738)/Homer	1e-3	-7.731e+00	0.0031	337.0	27.24%	11235.2	23.14%	motif file (matrix)	svg
53	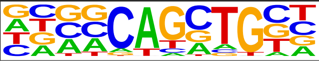	Ascl1(bHLH)/NeuralTubes-Ascl1-ChIP-Seq(GSE55840)/Homer	1e-3	-7.725e+00	0.0031	437.0	35.33%	14986.3	30.87%	motif file (matrix)	svg
54	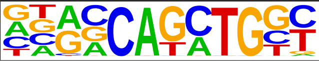	Atoh1(bHLH)/Cerebellum-Atoh1-ChIP-Seq(GSE22111)/Homer	1e-3	-7.218e+00	0.0049	320.0	25.87%	10687.2	22.02%	motif file (matrix)	svg
55	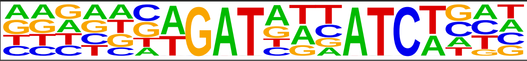	GATA(Zf),IR3/iTreg-Gata3-ChIP-Seq(GSE20898)/Homer	1e-3	-7.187e+00	0.0050	103.0	8.33%	2930.8	6.04%	motif file (matrix)	svg
56	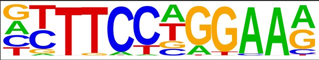	STAT4(Stat)/CD4-Stat4-ChIP-Seq(GSE22104)/Homer	1e-3	-6.922e+00	0.0064	375.0	30.32%	12790.4	26.35%	motif file (matrix)	svg
57	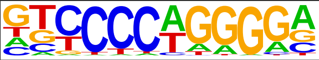	EBF1(EBF)/Near-E2A-ChIP-Seq(GSE21512)/Homer	1e-2	-6.821e+00	0.0070	258.0	20.86%	8466.8	17.44%	motif file (matrix)	svg
58	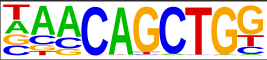	Tcf21(bHLH)/ArterySmoothMuscle-Tcf21-ChIP-Seq(GSE61369)/Homer	1e-2	-6.709e+00	0.0077	276.0	22.31%	9144.4	18.84%	motif file (matrix)	svg
59	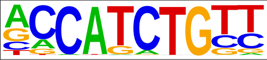	NeuroG2(bHLH)/Fibroblast-NeuroG2-ChIP-Seq(GSE75910)/Homer	1e-2	-6.689e+00	0.0077	469.0	37.91%	16395.3	33.77%	motif file (matrix)	svg
60	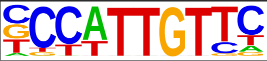	Sox2(HMG)/mES-Sox2-ChIP-Seq(GSE11431)/Homer	1e-2	-6.674e+00	0.0077	355.0	28.70%	12084.7	24.89%	motif file (matrix)	svg
61	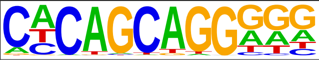	Unknown-ESC-element(?)/mES-Nanog-ChIP-Seq(GSE11724)/Homer	1e-2	-6.554e+00	0.0085	164.0	13.26%	5113.3	10.53%	motif file (matrix)	svg
62		ETV1(ETS)/GIST48-ETV1-ChIP-	1e-2	-6.547e+00	0.0085	380.0	30.72%	13046.2	26.88%	motif	svg

	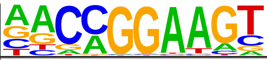	Seq(GSE22441)/Homer								file (matrix)	
63	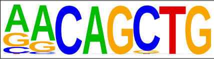	MyoG(bHLH)/C2C12-MyoG-ChIP-Seq(GSE36024)/Homer	1e-2	-6.505e+00	0.0086	283.0	22.88%	9435.3	19.44%	motif file (matrix)	svg
64	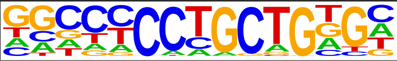	Zic3(Zf)/mES-Zic3-ChIP-Seq(GSE37889)/Homer	1e-2	-6.462e+00	0.0089	148.0	11.96%	4559.9	9.39%	motif file (matrix)	svg
65	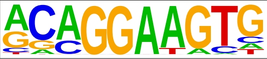	ETS1(ETS)/Jurkat-ETS1-ChIP-Seq(GSE17954)/Homer	1e-2	-6.293e+00	0.0104	301.0	24.33%	10137.5	20.88%	motif file (matrix)	svg
66	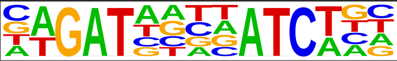	GATA(Zf),IR4/iTreg-Gata3-ChIP-Seq(GSE20898)/Homer	1e-2	-6.129e+00	0.0120	45.0	3.64%	1115.6	2.30%	motif file (matrix)	svg
67	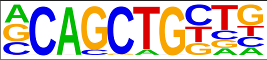	Tcf12(bHLH)/GM12878-Tcf12-ChIP-Seq(GSE32465)/Homer	1e-2	-6.116e+00	0.0120	256.0	20.70%	8504.7	17.52%	motif file (matrix)	svg
68	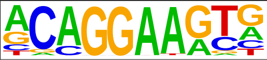	ERG(ETS)/VCaP-ERG-ChIP-Seq(GSE14097)/Homer	1e-2	-6.041e+00	0.0127	459.0	37.11%	16143.0	33.25%	motif file (matrix)	svg
69	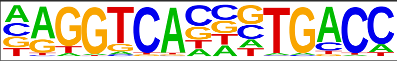	ERE(NR),IR3/MCF7-ERa-ChIP-Seq(Unpublished)/Homer	1e-2	-5.975e+00	0.0134	84.0	6.79%	2400.3	4.94%	motif file (matrix)	svg
70	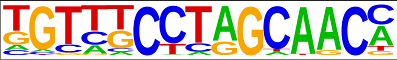	Rfx6(HTH)/Min6b1-Rfx6.HA-ChIP-Seq(GSE62844)/Homer	1e-2	-5.953e+00	0.0135	303.0	24.49%	10271.4	21.16%	motif file (matrix)	svg
71	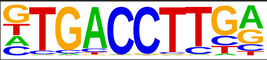	Esrrb(NR)/mES-Esrrb-ChIP-Seq(GSE11431)/Homer	1e-2	-5.888e+00	0.0142	213.0	17.22%	6967.3	14.35%	motif file (matrix)	svg
72	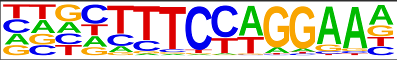	Bcl6(Zf)/Liver-Bcl6-ChIP-Seq(GSE31578)/Homer	1e-2	-5.765e+00	0.0159	451.0	36.46%	15895.8	32.75%	motif file (matrix)	svg
73	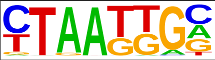	Isl1(Homeobox)/Neuron-Isl1-ChIP-Seq(GSE31456)/Homer	1e-2	-5.686e+00	0.0169	715.0	57.80%	26181.3	53.93%	motif file (matrix)	svg
74	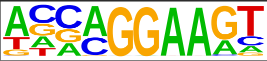	EHF(ETS)/LoVo-EHF-ChIP-Seq(GSE49402)/Homer	1e-2	-5.677e+00	0.0169	415.0	33.55%	14541.5	29.96%	motif file (matrix)	svg
75	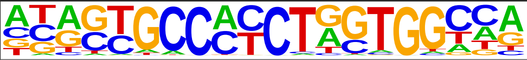	CTCF(Zf)/CD4+-CTCF-ChIP-Seq(Barski_et_al.)/Homer	1e-2	-5.358e+00	0.0229	32.0	2.59%	758.8	1.56%	motif file (matrix)	svg
76	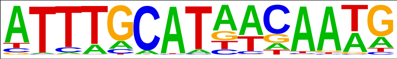	OCT4-SOX2-TCF-NANOG(POU,Homeobox,HMG)/mES-Oct4-ChIP-Seq(GSE11431)/Homer	1e-2	-5.299e+00	0.0239	129.0	10.43%	4032.3	8.31%	motif file (matrix)	svg
77	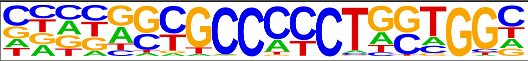	BORIS(Zf)/K562-CTCFL-ChIP-Seq(GSE32465)/Homer	1e-2	-5.202e+00	0.0260	49.0	3.96%	1304.3	2.69%	motif file (matrix)	svg
78	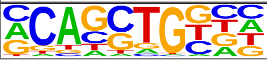	HEB(bHLH)/mES-Heb-ChIP-Seq(GSE53233)/Homer	1e-2	-5.161e+00	0.0268	568.0	45.92%	20542.5	42.32%	motif file	svg
										(matrix)	
79	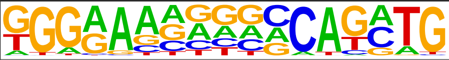	RBPJ:Ebox(?,bHLH)/Panc1-Rbpj1-ChIP-Seq(GSE47459)/Homer	1e-2	-5.104e+00	0.0280	102.0	8.25%	3107.6	6.40%	motif file (matrix)	svg
80	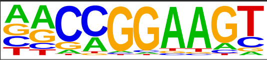	GABPA(ETS)/Jurkat-GABPa-ChIP-Seq(GSE17954)/Homer	1e-2	-5.102e+00	0.0280	244.0	19.73%	8235.8	16.97%	motif file (matrix)	svg
81	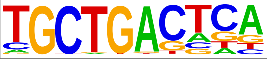	MafA(bZIP)/Islet-MafA-ChIP-Seq(GSE30298)/Homer	1e-2	-5.070e+00	0.0282	223.0	18.03%	7466.9	15.38%	motif file (matrix)	svg
82	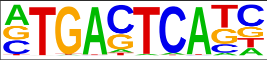	AP-1(bZIP)/ThioMac-PU.1-ChIP-Seq(GSE21512)/Homer	1e-2	-5.031e+00	0.0290	252.0	20.37%	8544.4	17.60%	motif file (matrix)	svg
83	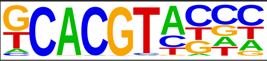	HIF2a(bHLH)/785_O-HIF2a-ChIP-Seq(GSE34871)/Homer	1e-2	-4.991e+00	0.0298	104.0	8.41%	3190.8	6.57%	motif file (matrix)	svg
84	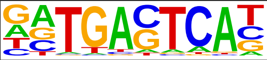	JunB(bZIP)/DendriticCells-Junb-ChIP-Seq(GSE36099)/Homer	1e-2	-4.911e+00	0.0319	191.0	15.44%	6320.7	13.02%	motif file (matrix)	svg
85	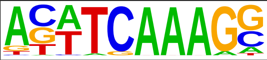	Tcf3(HMG)/mES-Tcf3-ChIP-Seq(GSE11724)/Homer	1e-2	-4.756e+00	0.0368	112.0	9.05%	3501.0	7.21%	motif file (matrix)	svg
86	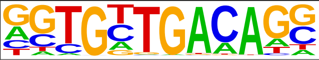	Tbx20(T-box)/Heart-Tbx20-ChIP-Seq(GSE29636)/Homer	1e-2	-4.755e+00	0.0368	72.0	5.82%	2109.3	4.35%	motif file (matrix)	svg
87	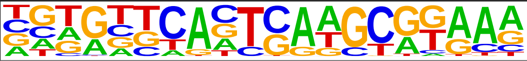	PAX6(Paired,Homeobox)/Forebrain-Pax6-ChIP-Seq(GSE66961)/Homer	1e-2	-4.686e+00	0.0386	38.0	3.07%	986.6	2.03%	motif file (matrix)	svg
88	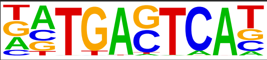	BATF(bZIP)/Th17-BATF-ChIP-Seq(GSE39756)/Homer	1e-2	-4.642e+00	0.0399	230.0	18.59%	7801.3	16.07%	motif file (matrix)	svg
