# Supplementary material for: TLE3 loss confers AR inhibitor resistance by facilitating GR-mediated human prostate cancer cell growth
Source: eLife. 2019 Dec 19;8:e47430. doi: 10.7554/eLife.47430 (PMC6968917; doi:10.7554/eLife.47430)
Supplement: Supplementary file 2. [file elife-47430-supp2.docx]

| **Key Resources Table** | | | | |
| --- | --- | --- | --- | --- |
| **Reagent type (species) or resource** | **Designation** | **Source or reference** | **Identifiers** | **Additional information** |
| gene (*Human*) | TLE3 |  | Gene ID: 7090 |  |
| gene (*Human*) | NR3C1 (GR) |  | Gene ID: 24413 |  |
| Cell line  (Human) | LNCaP | Kind gift from W. Zwart lab |  |  |
| Cell line  (Human) | LAPC4 | Kind gift from W. Zwart lab |  |  |
| Cell line  (Human) | CWR-R1 | Kind gift from W. Zwart lab |  |  |
| Cell line  (Human) | HEK | ATCC | CRL-1573 |  |
| Biological sample (*Homo-sapiens*) | Prostate tumor tissue | Biopsies from an in-house clinical trial |  | Collected pre- and post-enzalutamide treatment |
| Antibody | anti-TLE3  (mouse monoclonal) | Santa Cruz Biotechnology | #sc-514798 | 1:1000 Western blot  1:250 IHC |
| Antibody | anti-Vinculin  (rabbit monoclonal) | Sigma-Aldrich | #V4139 | 1:1000 Western blot |
| Antibody | anti-GAPDH  (rabbit monoclonal) | Cell Signaling Technology | #5174S | 1:10000 Western blot |
| Antibody | anti-GR  (rabbit monoclonal) | Cell Signaling Technology | #12041 | 7,5 μL/ChIP  1:600 IHC |
| Antibody | anti-H3K27ac  (rabbit polyclonal) | Active Motif | #39133 | 5 μg/ChIP |
| Recombinant DNA reagent | pLKO.1 Puro  (plasmid) | addgene | #10878 | shRNA backbone |
| Recombinant DNA reagent | LentiCRISPR 2.0 (plasmid) | addgene | #52961 | CRISPR backbone |
| Recombinant DNA reagent | psPAX2  (plasmid) | addgene | #12260 | Lentiviral packaging plasmid |
| Recombinant DNA reagent | pMD2.G  (plasmid) | addgene | #12259 | Lentiviral packaging plasmid |
| Recombinant DNA reagent | GeCKO Human CRISPR knockout pooled library | addgene | Zhang et al., 2014 addgene #1000000048 | Used for the CRISPR screen |
| Sequence-based reagent | Scrambled shRNA | In-house library | shRNA sequence | CCTAAGGTTAAGTCGCCCTCG |
| Sequence-based reagent | TLE3-1 shRNA | In-house library | shRNA sequence | CGCCATTATGTGATGTACTAT |
| Sequence-based reagent | TLE3-5 shRNA | In-house library | shRNA sequence | CCTATGGCTTGAACATTGAAA |
| Sequence-based reagent | GR-1 shRNA | In-house library | shRNA sequence | GTGTCACTGTTGGAGGTTATT |
| Sequence-based reagent | GR-2 shRNA | In-house library | shRNA sequence | TGGATAAGACCATGAGTATTG |
| Sequence-based reagent | Non-targeting gRNA | In-house library | gRNA sequence | ACGGAGGCTAAGCGTCGCAA |
| Sequence-based reagent | TLE3 gRNA -2 | Zhang et al., 2014 addgene  #1000000048 | gRNA sequence | TTGACCCTCACCCCCCGATG |
| Sequence-based reagent | TLE3 gRNA -8 | Zhang et al., 2014 addgene  #100000048 | gRNA sequence | CGGGCCAGTGAGAAGCACCG |
| Sequence-based reagent | AFF3_FW | This paper | RT-qPCR primer | GTCATCTCGTTGGAGTTCCCA |
| Sequence-based reagent | AFF3_RV | This paper | RT-qPCR primer | AGTGCCTCTCTTACTCTGCTG |
| Sequence-based reagent | CA13_FW | This paper | RT-qPCR primer | CGAGCACAACGGTCCTATTCA |
| Sequence-based reagent | CA13_RV | This paper | RT-qPCR primer | CTAAGTGGTCGGAGGGAAGAG |
| Sequence-based reagent | GAPDH_FW | This paper | RT-qPCR primer | AGCCACATCGCTCAGACAC |
| Sequence-based reagent | GAPDH_RV | This paper | RT-qPCR primer | GCCCAATACCGACCAAATCC |
| Sequence-based reagent | GNAI1_FW | This paper | RT-qPCR primer | GCTCAACCAAATTACATCCCGA |
| Sequence-based reagent | GNAI1_RV | This paper | RT-qPCR primer | TCGTAGTCACTCAGTGCTACAC |
| Sequence-based reagent | GUCY1A2_FW | This paper | RT-qPCR primer | ACCTCAGAATTAGCATCAACACC |
| Sequence-based reagent | GUCY1A2_RV | This paper | RT-qPCR primer | TGAGTGTCACATCGAAGCTGC |
| Sequence-based reagent | IGFBP5_FW | This paper | RT-qPCR primer | ACCTGAGATGAGACAGGAGTC |
| Sequence-based reagent | IGFBP5_RV | This paper | RT-qPCR primer | GTAGAATCCTTTGCGGTCACAA |
| Sequence-based reagent | NR3C1_FW | This paper | RT-qPCR primer | TGCCGCTATCGAAAATGTCTT |
| Sequence-based reagent | NR3C1_RV | This paper | RT-qPCR primer | GGGTAGGGGTGAGTTGTGGT |
| Sequence-based reagent | RND3_FW | This paper | RT-qPCR primer | CCCTCTCTTACCCTGATTCGG |
| Sequence-based reagent | RND3_RV | This paper | RT-qPCR primer | TGGCGTCTGCCTGTGATTG |
| Sequence-based reagent | TNFRSF19_FW | This paper | RT-qPCR primer | GACCTCAGCTCCACGAATATG |
| Sequence-based reagent | TNFRSF19_RV | This paper | RT-qPCR primer | CACCCCACAACCAAGAGTCG |
| Sequence-based reagent | UGT2B17_FW | This paper | RT-qPCR primer | TGTGTTGGGAATATTCTGACTA |
| Sequence-based reagent | UGT2B17_RV | This paper | RT-qPCR primer | AGGGGTTTGGCTGGTTTAC |
| Sequence-based reagent | RND3 locus_FW | This paper | ChIP RT-qPCR primer | AAATCTCTCCCACCCTCCTG |
| Sequence-based reagent | RND3 locus_RV | This paper | ChIP RT-qPCR primer | CCTTATCAGGAGCACCCTGT |
| Sequence-based reagent | GNAI locus_FW | This paper | ChIP RT-qPCR primer | CCACTGGATGTCCTTGTTCA |
| Sequence-based reagent | GNAI locus_RV | This paper | ChIP RT-qPCR primer | GCAACACCCTGGAAAAACAT |
| Sequence-based reagent | TNFRSF19 locus_FW | This paper | ChIP RT-qPCR primer | AGCAATCCTTGCTTTGTGCG |
| Sequence-based reagent | TNFSRF19 locus_RV | This paper | ChIP RT-qPCR primer | TGGAGTTGTGATGAGTGCCC |
| Sequence-based reagent | GR negative control_FW | This paper | ChIP RT-qPCR primer | TGTTTATCCTTCCAAGCAGCAGT |
| Sequence-based reagent | GR_negative control_RV | This paper | ChIP RT-qPCR primer | AAGGTTCCTGCCTATTCTCCAAC |
| Chemical compounds | Enzalutamide | Medkoo Biosciences | #201821 |  |
| Chemical compounds | Apalutamide | Medkoo Biosciences | #204420 |  |
| Chemical compounds | Mifepristone | Medkoo Biosciences | #201870 |  |
| Chemical compounds | Hydrocortisone | Sigma-Aldrich | H0888-G |  |
| Chemical compounds | R1881 | Sigma-Aldrich | R0908-10MG |  |
| Commercial assay, kit | Isolate II RNA Mini Kit | Bioline | BIO-52073 | RNA isolation kit |
| Commercial assay, kit | DNeasy Blood & Tissue Kit | Qiagen | #69504 | DNA isolation kit |
| Commercial assay, kit | SensiFAST cDNA Synthesis Kit | Bioline | BIO-65054 | cDNA synthesis kit |
| Commercial assay, kit | SensiFAST SYBR Lo-ROX Kit | Bioline | BIO-94050 | RT-qPCR kit |
| Software | MAGeCK | (Li et al., 2014) |  |  |
| Software | DESeq2 | (Love et al., 2014) |  |  |
| Software | Enrichr | (Chen et al., 2013) |  |  |
| Software | javaGSEA desktop application | <http://software.broadinstitute.org/gsea> |  |  |
| Software | DFilter | (Kumar et al., 2013) |  |  |
| Software | MACS peak caller version 1.4 | (Zhang et al., 2008) |  |  |
| Software | Prism - Graphpad | <https://www.graphpad.com/scientific-software/prism/> |  |  |
| Software | R version 3.4.4 | <https://www.r-project.org> |  |  |
