# Supplementary material for: TLE3 loss confers AR inhibitor resistance by facilitating GR-mediated human prostate cancer cell growth
Source: eLife. 2019 Dec 19;8:e47430. doi: 10.7554/eLife.47430 (PMC6968917; doi:10.7554/eLife.47430)
Supplement: Transparent reporting form [file elife-47430-transrepform.docx]

***eLife’s* transparent reporting form**

We encourage authors to provide detailed information *within their submission* to facilitate the interpretation and replication of experiments. Authors can upload supporting documentation to indicate the use of appropriate reporting guidelines for health-related research (see [EQUATOR Network](http://www.equator-network.org/%20)), life science research (see the [BioSharing Information Resource](https://biosharing.org/)), or the [ARRIVE guidelines](http://www.plosbiology.org/article/info:doi/10.1371/journal.pbio.1000412) for reporting work involving animal research. Where applicable, authors should refer to any relevant reporting standards documents in this form.

If you have any questions, please consult our Journal Policies and/or contact us: [editorial@elifesciences.org](mailto:editorial@elifesciences.org).

**Sample-size estimation**

- You should state whether an appropriate sample size was computed when the study was being designed
- You should state the statistical method of sample size computation and any required assumptions
- If no explicit power analysis was used, you should describe how you decided what sample (replicate) size (number) to use

Please outline where this information can be found within the submission (e.g., sections or figure legends), or explain why this information doesn’t apply to your submission:

At the time of the design of the experiments, the study was largely exploratory and effect size was unknown, meaning that sample size / power estimation was not feasible.

**Replicates**

- You should report how often each experiment was performed
- You should include a definition of biological versus technical replication
- The data obtained should be provided and sufficient information should be provided to indicate the number of independent biological and/or technical replicates
- If you encountered any outliers, you should describe how these were handled
- Criteria for exclusion/inclusion of data should be clearly stated
- High-throughput sequence data should be uploaded before submission, with a private link for reviewers provided (these are available from both GEO and ArrayExpress)

Please outline where this information can be found within the submission (e.g., sections or figure legends), or explain why this information doesn’t apply to your submission:

The CRISPR screen was performed using 3 biological replicates.

The RNA-seq experiment was performed using 3 biological replicates.

To review GEO accession GSE130246:

Go to <https://www.ncbi.nlm.nih.gov/geo/query/acc.cgi?acc=GSE130246>
Enter token gvqryumshdstzin into the box

qPCR experiments were performed at least 3 times (3 biological replicates) using 1 control gRNA/shRNA and 2 different gRNAs/shRNAs targeting the gene of interest. Within each experiment, 3 technical replicates were performed for each condition.

Western blots were performed at least 3 times (3 biological replicates).

Biological replicate: These were the independent experiments, the same test/experiment was performed using a different batch of cells infected with lentiviral construct (CRISPR or shRNA) at a different time.

Technical replicate: testing the same sample (biological replicate) multiple times (3 or more) within an experiment.

**Statistical reporting**

- Statistical analysis methods should be described and justified
- Raw data should be presented in figures whenever informative to do so (typically when N per group is less than 10)
- For each experiment, you should identify the statistical tests used, exact values of N, definitions of center, methods of multiple test correction, and dispersion and precision measures (e.g., mean, median, SD, SEM, confidence intervals; and, for the major substantive results, a measure of effect size (e.g., Pearson's r, Cohen's d)
- Report exact p-values wherever possible alongside the summary statistics and 95% confidence intervals. These should be reported for all key questions and not only when the p-value is less than 0.05.

Please outline where this information can be found within the submission (e.g., sections or figure legends), or explain why this information doesn’t apply to your submission:

Figure 1A and 1B, Figure 1-figure supplement 1C

For the CRISPR screen, the treated samples were compared with the untreated samples. A sgRNA was considered to be a hit, if the log2FC >= 3 and the FDR <= 0.1. TLE3 was the only gene for which all three sgRNAs were a hit. The MAGeCK (Li *et al*., 2014) analysis was done using the default settings, which produced TLE3 as top hit with a FDR of 0.002 and a p-value of 2.248E-07.

Figure 2A

For transcription factor enrichment analysis the list of differentially expressed genes were uploaded to Enrichr (Kuleshov *et al*., 2016) and the default settings were used generating indicated p-values.

Fig. 3B

ChIP binding for proteins at genes differentially expressed (DESeq2) in TLE3KO under enza treatment.

Figure 2D and 2E, Figure 2-figure supplement 1D

GSEA plots were generated using javaGSEA using the default settings resulting in indicated p-values.

qPCR experiments (Figure 2C, Figure 4E, Figure 1-figure supplement 1D, Figure 2-figure supplement 1C

Error bars represent the average data of at least 3 independent experiments (biological replicates) using 3 technical replicates within each experiment (± SEM). P-values are indicated with ****P* < 0.001, ***P* < 0.01 and **P* < 0.05 (two-tailed *t*-test).

Figure 3-figure supplement 1A

Wilcoxon test comparing log fold change of differentially expressed genes with and without TLE3 binding.

(For large datasets, or papers with a very large number of statistical tests, you may upload a single table file with tests, Ns, etc., with reference to sections in the manuscript.)

**Group allocation**

- Indicate how samples were allocated into experimental groups (in the case of clinical studies, please specify allocation to treatment method); if randomization was used, please also state if restricted randomization was applied
- Indicate if masking was used during group allocation, data collection and/or data analysis

Please outline where this information can be found within the submission (e.g., sections or figure legends), or explain why this information doesn’t apply to your submission:

n/a

**Additional data files (“source data”)**

- We encourage you to upload relevant additional data files, such as numerical data that are represented as a graph in a figure, or as a summary table
- Where provided, these should be in the most useful format, and they can be uploaded as “Source data” files linked to a main figure or table
- Include model definition files including the full list of parameters used
- Include code used for data analysis (e.g., R, MatLab)
- Avoid stating that data files are “available upon request”

Please indicate the figures or tables for which source data files have been provided:

-Figure 1-source data 1: Readcounts CRISPR resistance screen

-Figure 1-source data 2: Processed data (DESeq2 analysis output)

-Figure 1-source data 3: Processed data (MAGeCK analysis output)

-Figure 2-source data 1: Readcounts RNA-seq for control and TLE3KO cells treated with vehicle or 10 uM enzalutamide for 5 days
